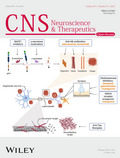# Front cover

**DOI:** 10.1111/cns.14374

**Published:** 2023-07-18

## Abstract

The cover image is based on the Review Article *Advances and applications of fluids biomarkers in diagnosis and therapeutic targets of Alzheimer's disease* by Yanan Xu et al., https://doi.org/10.1111/cns.14238.